# *“I will find the best method that will work for me”*: navigating contraceptive journeys amongst South African adolescent girls and young women

**DOI:** 10.1186/s40834-024-00298-4

**Published:** 2024-08-02

**Authors:** Zoe Duby, Kate Bergh, Brittany Bunce, Kim Jonas, Nevilene Slingers, Catherine Mathews, Fareed Abdullah

**Affiliations:** 1 Health Systems Research Unit, South African Medical Research Council, Cape Town, South Africa; 2 School of Public Health, University of Cape Town, Cape Town, South Africa; 3 Universitat Autònoma de Barcelona, Barcelona, Spain; 4 Department of Psychology, University of Cape Town, Cape Town, South Africa; 5 Office of AIDS and TB Research, South African Medical Research Council, Pretoria, South Africa; 6 Department of Public Health Medicine, Faculty of Health Sciences, University of Pretoria, Pretoria, South Africa; 7 Division of Infectious Diseases, Department of Internal Medicine, Faculty of Health Sciences, University of Pretoria, Pretoria, South Africa

**Keywords:** Contraceptive journeys, South Africa, Adolescent girls and young women, Contraceptive methods, Contraceptive decision-making

## Abstract

**Background:**

Given that South Africa has one of the highest rates of pregnancy amongst adolescent girls and young women (AGYW) globally, the provision of contraceptives to this group has been a key focus in recent years. Pregnancy prevention involves an on-going continuum of decision-making around contraceptive method choice, uptake, use, experience, continuation, and discontinuation.

**Methods:**

This paper presents analysis of data from a cross-sectional survey with 2376 AGYW, as well as qualitative in-depth interviews (IDIs) with 54 AGYW, inclusive of contraceptive journey narratives. We examine the preferences, valued characteristics, choices, beliefs, understandings and experiences of choosing and using contraceptives amongst AGYW in two South African communities characterised by high rates of pregnancy.

**Results:**

These findings shed light on the preferences towards, beliefs about, and experiences of choosing, using and discontinuing contraceptive methods amongst this population, with survey data suggesting that the most popular methods were the injection, followed by the implant, and then the oral pill. Findings illustrate the complexity and dynamic nature of contraceptive decision-making and the varied embodied and lived experiences of contraceptive use, and how these are impacted by contraception service provision.

**Conclusions:**

Our findings show that contraception experiences of each individual are cumulative, and comprise a continuum of method initiation, use, discontinuation, method switching and on-going circular decision-making influenced by multiple social, structural, contextual and interpersonal factors, combined with shifting preferences, values and needs. To maximise the use of contraceptives amongst South African AGYW, it is necessary to provide responsive contraception service provision to reflect the changing contexts and preferences of users, in order to ensure that pregnancy prevention needs are catered for throughout their reproductive life course.

## Introduction

There has been an increasing volume of research examining the ‘unmet need’ for contraceptives, in acknowledgement of the various circumstances and factors that hinder and constrain contraceptive use [1]. In South Africa, the rates of pregnancy amongst adolescent girls and young women (AGYW) are amongst the highest in the world, and national data suggests that ‘unmet need’ for contraceptives among youth under 25 years old is around 30% [2]. Research assessing South African AGYW’s access to contraceptives, and exploring barriers to access has shown that access to, and motivation to use contraceptives are lower amongst adolescent girls than among older women [3]. Whilst increasing uptake of, and reducing barriers to access of contraceptives for AGYW is critical, it is also necessary to consider the continuum of uptake, use, and discontinuation, and women’s on-going decision-making around pregnancy prevention, as well as the social, structural, contextual and interpersonal factors that influence decision-making over the reproductive life course. Global reviews suggest that contraceptive users’ values and preferences are context specific, and vary across settings, population groups, geographic areas, and socio-cultural communities [4].

There is currently relatively scant data available on AGYW decision-making around when to use a contraceptive, which contraceptive method to choose, use, and continue using, as well as evidence to inform an understanding of the lived realities and subjective experiences of AGYW as they navigate their contraceptive journeys. It is not only the decision made at a singular point of time, regarding whether or not to use contraceptives that matters, but also which method to choose at any given time. The factors that influence the decision-making process, the subsequent experiences of using the chosen contraceptive method, and decisions to stop using that method or switch to another are critical to understand in order to provide the best contraceptive care, and support AGYW on their contraceptive journeys [5, 6]. Existing research related to South African women’s perceptions of modern contraceptives has not fully considered the embodied experiences and lived realities of women throughout the process of navigating, choosing, and using different contraceptive methods; gaining an insight into these can help to inform responsive and appropriate contraceptive services [7].

Evidence shows that increasing the variety of contraceptive methods available to choose from, increases overall contraceptive use [8]. Currently, ‘modern contraceptive methods’ recommended globally comprise of long-acting reversible contraceptives (LARCs), inclusive of intrauterine devices (IUDs/IUCDs) and subdermal implants, and short-acting methods such as injectables and the oral daily pill. It has been suggested that amongst AGYW globally, LARCs are not only safe to use, but are preferable to short-acting contraceptives, demonstrating higher efficacy, continuation rates, and user satisfaction as compared to short-acting methods [8, 9]. Proponents of LARCs argue that access to these methods for AGYW will ensure more reliable, cost effective, convenient and longer term protection from unwanted pregnancies as compared to short-acting contraceptives and coitally-dependent methods such as condoms or emergency contraception [5].

In theory, South African AGYW aged 12 years and upwards, are able to access a wide range of contraceptives without parental consent, and free of charge, at government health facilities [10]. Methods that should be accessible to them include daily oral pills/tablets, injectable contraceptive agents (three-monthly injectable Depo-Provera^®^ and two-monthly injectable Nur-Isterate^®^), subdermal implants (Implanon^®^), and barrier methods such as male and female condoms. However, evidence suggests that AGYW are rarely provided with comprehensive counselling and information about all the contraceptive methods available, and details on their respective side effects and mechanisms of action [11, 12]. In reality, despite a range of contraceptives being ‘available’ to AGYW, there are numerous barriers to AGYW contraceptive access, and evidence suggests that AGYW are generally offered fewer contraceptive method options than older women [11]. The reproductive justice framework outlines that respect for an adolescent’s right to choose or decline any method of contraception from a range of high quality, safe, effective, and acceptable methods, based on informed decision-making, and free from coercion, should be paramount [9, 13].

In this paper we employ the term ‘contraceptive journey’, to recognise that contraceptive use is not a once-off intervention, but rather an on-going iterative process of decision-making and embodied experience that plays out over several decades in the life of a person with reproductive capacity [6, 14]. In recent literature there has been a shift towards this framing, with the proposition of regarding contraceptive use as a multi-phase continuum comprising deciding to prevent pregnancy, choosing and accessing a method, initiating use, using, and discontinuing use [6, 15]. At each of these phases on the continuum, decisions are made based on a variety of factors inclusive of somatic and lived experiences, perceptions of efficacy, context, interpersonal relationships and perceptions of the views of others [6].

There has been limited research focusing on the views and experiences of adolescent girls and young women in South Africa in relation to their contraceptive decision-making and pregnancy prevention journeys. A deeper understanding of the preferences, valued characteristics, perceptions, and embodied experiences of AGYW towards contraceptives will enable service provision to be more responsive to the ‘unmet need’ [4]. In this analysis, we set out to examine the preferences, valued characteristics, choices, understandings and experiences of choosing and using contraceptives amongst AGYW in two South African communities characterised by high rates of pregnancy amongst this age group. Using the narratives of AGYW, we seek to describe the ways in which they navigate their contraceptive journeys. In this paper, we consciously avoid the term ‘family planning’, and instead refer to contraceptive use. Although the term ‘family planning’ is commonly used as a synonym for the use of contraception, we believe that this terminology privileges the concept of creating a family as being the paramount ‘goal’ in a woman’s sexual and reproductive life course, framing her sexual and reproductive health (SRH) decision-making within a traditional, heteronormative biological idea [16].

## Methods

The analysis presented in this paper comprises data from a quantitative survey and qualitative interviews that formed the pre-intervention component of a study evaluating a combination SRH intervention for AGYW in South Africa. The intervention provides a range of clinical sexual and reproductive health services, psycho-social support services, and social structural services to AGYW in six schools in Moretele in the North West (NW) province and eight schools in Newcastle in KwaZulu-Natal (KZN). Details of the evaluation and programme are available in Bergh et al. 2023 [17]. All AGYW who took part in the survey and interviews provided informed consent for participation. For those under 18 years of age, consent from a parent or legal guardian was obtained first. Participants received R150 (US$ 8) reimbursement for each interview they participated in. Ethical approval to conduct this study was obtained from the Human Research Ethics Committees at the South African Medical Research Council, and the University of Cape Town (EC045-10/2021).

### Quantitative methods

A cross-sectional baseline survey was conducted between July and September 2022, among AGYW aged 13 years and older in grades 9–12 in all 14 programme schools in Moretele and Newcastle. Two to three classes were randomly sampled per grade to ensure a minimum of 40 participants per grade. This allowed for a total expected sample of 2240 AGYW, to ensure the study had more than 80% statistical power to measure desired changes in primary outcomes at the follow-up survey, based on a pair-matched study design.

Among all survey participants, we report on socio-demographic characteristics including AGYW’s age, the site from which they were sampled, their relative socio-economic status, and whether they had ever been pregnant or planned to become pregnant within the year following the survey. Relative socio-economic status was created using cluster analysis with 13 variables relating to household assets; having, saving and owing money; and food insecurity [18]. We also report on AGYW’s attitudes towards three different contraceptive methods: (1) the injection, (2) the implant and (3) the oral daily pill. Among AGYW who had ever used a modern contraceptive method, we report on the quality of care received by AGYW at their last visit to a clinic, hospital or health worker to get contraceptives.

### Qualitative methods

**Table 1:** Qualitative sample

Adolescent Girls and Young Women (AGYW)	Total	Moretele, North West	Newcastle, KwaZulu-Natal
**AGYW 15–24 years** AGYW 15–18 years AGYW 19–24 years	**54** 41 13	**19** 15 4	**35** 26 9

In-depth interviews (IDIs) were conducted with a total of 54 AGYW between aged of 15 and 24, comprising 41 AGYW aged between 15 and 18 years, and 13 AGYW aged between 19 and 24 years (Table 1). All interviews were conducted by two female interviewers fluent in the local languages: Setswana and Sesotho in Moretele, and isiZulu in Newcastle. Interviews followed semi-structured topic guides, were conducted in participants’ language of choice, and audio-recorded with participants’ consent.

### Data analysis

#### Quantitative analysis

Frequencies, proportions and confidence intervals are reported. Results were adjusted for the multistage study design, which included the following stages: (1) schools stratified by district; (2) classes stratified by grade and (3) individual students.

#### Qualitative analysis

Audio recordings of interviews were transcribed and translated into English. Transcripts were analysed using an iterative analytical process in which four data analysts collaboratively organised and coded data. Initially, predetermined deductive code type based on the topics included in the interview guides were used as a preliminary framework. Using a cyclic and iterative process, codes and themes were built upon through inductive development and refinement. Analysts collaboratively worked on evolving analytical memos, capturing emergent themes. Two of the analysts worked collaboratively to interpret the coded data. The contraceptive journey narratives underwent a member checking process, in which the participants were invited to review and give feedback on their own narratives, to ensure accuracy in representation of their experiences, and comment on whether the narratives were a reasonable reflection of their experiences [19, 20].

## Findings

### Quantitative findings

Table 2 describes the socio-demographic characteristics of all 2376 AGYW survey participants. Of these AGYW, 76.4% were in the 13–17 age group and 23.6% were in the 18–23 age group. Keeping in mind that 6 schools in Moretele (NW) and 8 schools in Newcastle (KZN) were involved in the Imagine Programme, 43.6% of participants were from Moretele and 56.4% were from Newcastle. Less than half of participants were in the relatively low socio-economic status group (39.6%). Overall, 10.6% of participants had ever been pregnant and 8.6% had ever given birth to a child; both of these variables were, unsurprisingly, statistically significantly higher in the older age group compared to the younger age group. Very few of this school-going sample of AGYW had plans to become pregnant within the year after the survey in the younger (0.7%) or older age group (1.2%) (Table 2).

As shown in Table 3, all 2376 AGYW survey participants reported on their attitudes towards three different modern contraceptives: the injection, the implant and the daily oral pill. Less than half of all participants thought that the injection was safe to use (45.2%), the pill was safe to use (36.9%) and the implant was safe to use (30.1%). The injection was viewed as the best method to prevent pregnancy (61.6%) compared to the implant (46.2%) and pill (42.1%). Although the injection was viewed as the safest of the three modern contraceptives and the best way to prevent pregnancy, more than half of participants believed that the injection makes your body change in unpleasant ways (53.2%). This is in contrast to 32.1% of participants who reported that the implant causes irregular bleeding and 28.2% of participants who reported that the pill makes your body change in unpleasant ways. In addition, there were significantly fewer participants in the 13–17 age group (59.5%) who thought that the injection was a good method to prevent pregnancy compared to the 18–24 age group (68.6%).

Of the 2376 survey respondents, only 21.3% (*n* = 507) reported that they had ever used a modern contraceptive method. Of these participants, 52.9% were in the younger age group and 47.1% were in the older age group. Table 4 describes the quality of care reported by AGYW at their last visit to a clinic, hospital or health worker to get contraception among participants who had ever used a modern contraceptive method. Among these participants, 50.3% of participants reported that the health worker checked whether they were happy with the contraceptive method that they had been using. Most AGYW (68.2%) reported that they had been told about the injection, 53.1% were told about the implant, 43.2% were told about the pill, 30.2% were told about condoms, and very few participants were told about the IUD (5.7%) and emergency contraception (5.5%). Most AGYW (71.8%) were asked which contraceptive method they would like most, while 22.9% reported that they were steered or pushed towards a specific contraceptive method. Among AGYW who reported that they were steered or pushed towards a specific method (*n* = 116), 58.6% reported that they were steered or pushed towards the injection, 29.3% were steered towards the implant, 5.2% were steered towards condoms and 4.3% were steered towards the pill. There were no statistically significant differences by age group.

**Table 2:** Socio-demographic characteristics of baseline survey participants (*n* = 2376)

Socio-demographic characteristic	13–17 age group		18–24 age group		Total	
	Freq (%)	95% CI*	Freq (%)	95% CI	Freq (%)	95% CI
Site						
Moretele	801 (44.1)	41.4–46.8	236 (42.1)	35.0-49.6	1037 (43.6)	42.1–45.1
Newcastle	1016 (55.9)	53.2–58.6	324 (57.9)	50.4–65.0	1340 (56.4)	54.9–57.9
Relatively low socio-economic status**	639 (38.5)	32.9–44.5	229 (42.8)	35.0–51.0	868 (39.6)	34.2–45.2
Ever been pregnant	101 (5.6)	4.8–6.4	151 (27.0)	23.3–30.9	252 (10.6)	8.9–12.6
Given birth to a child	81 (4.5)	3.5–5.6	124 (22.1)	18.8–25.9	205 (8.6)	6.9–10.7
Planning to become pregnant within the next year	12 (0.7)	0.4–1.2	7 (1.2)	0.6–2.7	19 (0.8)	0.5–1.3

*95% confidence interval; **Relative socio-economic status has 184 missing observations as this variable was created from 13 variables through a cluster analysis that cannot include missing data.

**Table 3:** AGYW attitudes towards different contraceptive methods (the injection, implant or daily oral pill) based on baseline survey responses (*n* = 2376)

AGYW agrees or strongly agrees with the following statements	13–17 age group		18–23 age group		Total	
	Freq (%)	95% CI*	Freq (%)	95% CI	Freq (%)	95% CI
It is safe for a young woman like me to use the injection	785 (43.2)	39.8–46.7	289 (51.6)	46.3–56.9	1074 (45.2)	41.7–48.7
The injection is a good method to prevent pregnancy for young woman like me	1080 (59.5)	56.7–62.1	384 (68.6)	62.7–73.9	1464 (61.6)	58.8–64.4
The injection makes your body change in unpleasant ways	937 (51.6)	48.8–54.4	328 (58.6)	53.2–63.7	1265 (53.2)	50.7–55.8
It is safe for a young person like me to use the implant	506 (27.9)	26.0-29.8	208 (37.1)	32.4–42.2	714 (30.1)	28.2–32.0
The implant is a good method to prevent pregnancy for young woman like me	815 (44.9)	42.2–47.6	283 (50.5)	45.6–55.5	1098 (46.2)	43.7–48.7
The implant causes irregular bleeding	515 (28.4)	26.1–30.7	247 (44.1)	39.7–48.7	762 (32.1)	30.6–33.6
Using the implant makes it difficult to fall pregnant when it is removed	447 (24.6)	21.8–27.7	149 (26.6)	24.3–29.1	596 (25.1)	22.9–27.4
The pill is a good method to prevent pregnancy for young women like me	746 (41.1)	37.8–44.5	255 (45.5)	41.7–49.4	1001 (42.1)	39.2–45.2
The pill is safe for a young woman like me	644 (35.5)	32.2–38.9	232 (41.4)	36.6–46.4	876 (36.9)	34.2–39.6
The pill makes your body change in unpleasant ways	520 (28.6)	25.8–31.7	150 (26.8)	22.2–31.9	670 (28.2)	25.6–31.0

*95% confidence interval

**Table 4:** Quality of care reported by AGYW at their last visit to a clinic, hospital or health worker to get contraception among AGYW who have ever used a modern contraceptive method based on baseline survey responses (*n* = 507)

Quality of care measures	13–17 age group		18–23 age group		Total	
	Freq (%)	95% CI*	Freq (%)	95% CI	Freq (%)	95% CI
Health worker checked if AGYW was happy with contraceptive method she had been on before	135 (50.4)	44.9–55.9	120 (50.2)	42.6–57.8	255 (50.3)	46.2–54.4
AGYW was told about the pill	114 (42.5)	34.8–50.6	105 (43.9)	35.6–52.7	219 (43.2)	37.2–49.4
AGYW was told about the injection	186 (69.4)	62.0-75.9	160 (66.9)	56.1–76.2	346 (68.2)	61.2–74.5
AGYW was told about the implant	136 (50.7)	43.1–58.4	133 (55.6)	48.7–62.4	269 (53.1)	49.1–57.0
AGYW was told about the intrauterine device (IUD)	16 [6]	3.4–10.4	13 (5.4)	3.4–8.6	29 (5.7)	4.5–7.3
AGYW was told about emergency contraception	19 (7.1)	4.0-12.3	9 (3.8)	2.0-7.1	28 (5.5)	3.7–8.1
AGYW was told about condoms	86 (32.1)	23.5–42.1	67 [21]	19.5–38.5	153 (30.2)	23.7–37.5
AGYW was told about another contraceptive method	10 (3.7)	1.2–10.8	8 (3.3)	1.4-8.0	18 (3.6)	1.8–6.9
AGYW was not told about different contraceptive methods	15 (5.6)	3.2–9.5	26 (10.9)	7.0-16.6	41 (8.1)	5.6–11.5
AGYW was asked which contraceptive method she would like most	188 (70.1)	62.7–76.7	176 (73.6)	66.1–80.0	364 (71.8)	67.1–76.1
AGYW was steered or pushed towards a specific contraceptive method	64 (23.9)	17.9–31.1	52 (21.8)	17.1–27.2	116 (22.9)	18.6–27.8

*95% confidence interval

### Qualitative findings

In the section below, we include a summary of generalised themes that emerged in the interviews with all 54 respondents in the qualitative sample. The narrative journeys presented come from a sub-sample of AGYW who participated in multiple interviews, selected based on their self-reporting and disclosure of contraceptive use, and willingness to discuss their contraceptive decision-making processes and journeys. All names presented alongside data are pseudonyms.

#### Implant

Interview respondents shared their views of, and experiences using the subdermal implant (Implanon^®^). Views on the implant were varied. Respondents explained that they receive mixed messaging, with many AGYW peers speaking negatively of the implant but health workers speaking positively of it. Respondents explained that it can be confusing receiving conflicting advice, and deciding who to listen to. The key motiving factor to use the implant described related to it being long-lasting, reflective of AGYW’s general reluctance to go to health facilities. It was a common narrative that fear of clinics was a barrier to choosing the injection, and motivation for choosing the implant, which requires less frequent clinic visits.

A common view expressed by AGYW was that the implant has a bad reputation, particularly relating to alleged side effects. In the qualitative interviews, respondents listed various side effects they had heard being attributed to the implant, including hunger and food cravings, heavy bleeding, body shape changes, spotting, irregular or cessation of periods, infertility and damage to the uterus. Additionally, AGYW cited fear of implant theft – a commonly held concern that ‘criminals’ accost women and forcibly remove their implants to use as a recreational substance. It was also suggested that the implant is an invasive and alien product inserted into the body. It was also suggested that for those who are slim/skinny, the implant would be visible under the skin. Other reasons the implant was not considered a good option for AGYW included the assertion that AGYW are unlikely to observe the waiting time with the implant before having unprotected sex. Some of the respondents who had personal experience with the implant explained that they had felt side effects including headaches, thirst, increased hunger, weight gain and disruptions to the menstrual cycle. Interruption to, or disruption of the menstrual cycle raised particular concern amongst AGYW. Few respondents spoke positively of their personal experiences using the implant, although both Nomsa’s and Lebo’s narrative journey below illustrate decision-making to continue using an implant.

#### Injectables

When speaking of injectable contraceptives, respondents referred to “2-month” and “3-month”. These refer to two-monthly injectable (Nur-Isterate^®^), or three-monthly injectable (Depo-Provera^®^ or Petogen). Several perceived positive attributes of injectables were described, including safety and efficacy, the belief that injectables have fewer side effects than the implant and are better for first time users of contraceptives. The key perceived benefit of injectables as compared to daily oral tablets was convenience, with the injection seen as more suitable for AGYW who are ‘busy’ and will forget to take tablets. It was also suggested that being healthcare provider administered, injections are safer, as you can’t ‘overdose’ as you can with tablets. Some believed that injectables could reduce period pains. However, there were some negative views of injectables as well, including fear of side effects, such as impacting negatively on libido and the menstrual cycle, and causing long term infertility.

In terms of views on injectables from AGYW who had personal experience using them, there were several positive accounts. There were cases in which AGYW had heard about potential side effects of injectables but had not experienced any themselves, such as Simphiwe, who said that she had not faced any challenges and plans to continue using the injectable:

*I did not have any side effects (from Depo)… I have not experienced what other people have said about it*, *like gaining or losing weight and having so much appetite… (for me) everything was normal… I will continue using it because… it is not giving me any challenges. (Newcastle*, *13–17 years)*

However there were several AGYW who reported that they had experienced side effects from using injectables, including changes in menstruation, which created mistrust in the method. Some AGYW who had experienced side effects of their contraceptive method said that they feel unable to report their dissatisfaction, being too shy to disclose them, and instead opt to discontinue without informing a healthcare provider. In the quotation below, Fundiswa explains her decision-making to choose the injectable, which she is still using, but describes the challenges she has faced, and her plan to discontinue:

*I am using Depo… (I chose this method because) I won’t have to go to the clinic regularly… I’ll forget to take tablets. I am a slender so I will not be able to put the implant because it will be visible. It is better to take this injection… (but) after next year I will stop using it… because there are side effects… I did not ask (the healthcare provider) about the side effects but I can see changes in my body. I don’t have a problem with gaining weight but the main challenge is the change in the menstruation cycle… It was not easy (at first) but now I am fine… although I am not trusting these injections. I menstruated for two weeks*, *so I do not trust them… There is water that comes from my vagina so I have to wear a pad all the time. Another thing is that I do not have feelings (loss of libido)… I have not said anything (reported side effects)… I am not yet comfortable to talk about that. (Newcastle*, *13–17 years)*

### Oral contraceptives

In the qualitative interviews, some AGYW believed the oral daily pill to be the best option for AGYW, for reasons including the fact that tablets are more familiar, and less scary than injections and implants, with fewer side effects. It was also stated that you can access the pill at a pharmacy if you want to avoid clinics, and you can stop using it at any time without having to go to a healthcare provider. It was suggested that pills ‘clean’ the body, removing the ‘dirt’. A perceived positive attribute of the pill was that since it does not cause a cessation in menstrual bleeding like the other methods, your ‘womb’ would be kept ‘clean’. Thandeka’s account below illustrates a positive experience using oral contraceptives, which she plans to continue using:

*I chose tablets… I am afraid of other methods like implant*, *and injections… Sometimes those methods cause changes in your menstrual cycle*, *but it depends on your body. Some people are not happy about the side effects… I never miss my tablets*, *I have even set the times… The nurse told me that I might get dizzy at first but things will get back to normal… They do say that it depends on the body system of the person… some lose appetite*, *but I am fine… I will continue with this method… the tablets are not giving me any challenges and I don’t know what these other methods will do to my body. (Newcastle*, *13–17 years).*

However, there were some reasons not to choose the pill listed by respondents. The main reason being that it is too easy to forget and then risk getting pregnant. Additionally, potential side effects such as ‘damage to the womb’, weight gain, acne and nausea, were also described. One additional concern regarding the pill related to fear of HIV stigma and assumptions being made if someone saw you taking a tablet, and mistook it for antiretroviral HIV treatment.

### Intrauterine devices (IUDs)

In the interviews, AGYW generally lacked information on IUDs, or “the loop”, which was regarded as a method more appropriate for older women. It was mentioned that like implants, IUDs also have the benefit of being long-lasting. However, in general AGYW voiced negative views on the IUD, fearing it could get displaced internally, the belief that it is not very effective, and if one gets pregnant using an IUD then the baby will be born with the IUD embedded in them. AGYW also disliked the way IUDs are inserted into the vagina, and found this invasive. None of the respondents had personal experiences using an IUD.

## Contraceptive journey narratives

### Nomsa’s contraceptive journey

Nomsa, a young woman aged 22, from Newcastle, narrates how her contraceptive journey began after giving birth to her first child, “it was compulsory to get contraceptives before leaving the hospital^1^, so you had to choose the method you will use… They wrote a letter of referral to the nearest clinic where you will get your contraceptives… they gave me the three months’ injection”. Nomsa describes the side effects she experienced after receiving the injection: “it was not good for my body… I felt dizzy, had terrible headache, menstruated for a long time and lost weight”. Nomsa used to “menstruate a lot”, and was given pills to mitigate the heavy bleeding. “I lost weight because of losing so much blood… I was bleeding too much”. Nomsa expresses an awareness that not everyone suffers the same side effects from using the injectable: “my friends did not have any problems with injections… I would not say it is bad (as a method) because others do not have problems with the injection, it is good for them and they have used it for a long time”. Nomsa explains that she decided “the injection wasn’t good for me so I decided to stop using it” however as result “I got pregnant because I was not using any contraceptives at that time”.

After having her second child, the next contraceptive method that Nomsa tried was the implant, even though she’d heard negative things about it from friends: “I used to hear people saying bad things about the implant… we (young women) do not want to use the implant because of what friends are saying about it. I decided not to listen to friends”. Nomsa explains that she chose the implant for its convenience: “I am using the implant because it lasts for years not months… sometimes you get lazy to go to the clinic to get the injection but the implant is there in your body. Sometimes you forget to take the pills and the injections… There is no one who influenced me (to choose implant), it was my idea because I had my first child while I was a teenager so I used an injection, but on top of that I got the second child so I decided to use the implant because they give it credit (say good things about it) at hospitals”.

Nomsa did experience some initial side effects after getting the implant: “at first I used to have headaches but my body got used to it. I was not supposed to use painkillers due to the headache. I used to drink lot of water when I had that headache.” She also experienced increased appetite: “another thing with the implant, it made me hungry… you eat a lot as if you are using drugs”. Her increased appetite presented a challenge to her financially constrained household: “Here at home they were surprised about my eating, I was eating as if I was pregnant… When the situation is not well here at home financially, I lose weight because I do not eat the way I am supposed to eat… But I have to get used to the situation, if the food is not enough there is nothing I can do”.

The other main side effect that Nomsa had using the implant was that she experienced heavy bleeding: “this is second year since I had the implant, but I still bleed like I used to do when I was using the injection”. In Nomsa’s view, the heavy bleeding is an indication of her fertility: “That means I have lot of blood, and there is nothing I can do to control the blood. They told me at the clinic that this means that I might have lot of children. They told me… that they cannot stop this blood because it might be dangerous to do that so I must continue to use these available contraceptive methods”.

Despite the side effects she’s experienced, and the bad things people say about the it, Nomsa’s decision is to keep using the implant, as it has been effective in preventing pregnancy, and is preferable to the injection, which she’d experienced side effects using: “I prefer the implant… It is good for me, because this is the second year and I am not pregnant, hoping that maybe it will be good until next year which will be a third year… People are saying different things about the implant but for me it is the second year… it is still good for me”. Nomsa added a statement on her rationale: “I will never use it (injection) again, if the implant is still good for me I will continue with it… the injection wasn’t good for me.”

In concluding, Nomsa explains how important peers are in choosing a contraceptive method: “We (young women) do not take contraceptives, we listen to bad advice from friends, if the friend is not happy about implant she will tell me not to take it also, she will tell me that I can get pregnant if use the implant. Friends will tell me the disadvantages of different types of contraceptive so you will end up deciding not to use them… the government has tried all means to give us protective measures but we do not use them and end up getting pregnant because we listen to friends… We end up not going to the clinic to take contraceptives because friends said that they are not 100% safe.”

### Vuyo’s contraceptive journey

In narrating her contraceptive journey, Vuyo, who is 24 years old and from Newcastle, explained: “I started using contraceptives after I had my child… The first reason is that I did not want to get pregnant and the second reason is that men do not want to use condoms”. Vuyo narrates her decision-making process: “I went to the clinic after giving birth… to get contraceptives… the nurse gave me information about types of injections… I chose 3 month because I thought it will work better for me”. She specifically declined the 2 month injection due to having heard negative things about it: “Due to those stories I have heard… they said that if you are using this (2 month) injection, sometimes you bleed for a long time, others said that the blood becomes dark. Some do not menstruate and others complain of headaches”. Based on what she’d heard about the different methods, she decided on the method she believed would best suit her: “I do not have much blood, sometimes I menstruate for one day or two… so I chose to use a 3 month injection… I heard from others that this method is good… many people were praising it… so I decided to try it”. She was warned by the healthcare provider that she may experience some side effects: “they gave me a 3 month injection… they told me to come back to the clinic if maybe I bleed too much then they will give me the tablets to stop bleeding”.

However, Vuyo explains that despite using the injection, she got pregnant: “I got pregnant whilst I was still using the injection… I think I failed to follow the instructions because they said we must wait for seven days before having sex after you have taken the injection”. After giving birth, Vuyo decided to get another 3 month injection in order to prevent another pregnancy. However she experienced unpleasant side effects: “It (injection) gives headaches, you feel dizzy and the body gets tired as if you can sleep… It (headache) is always there… I was always tired and stopped menstruating”. Vuyo heard that because the injection stops menstruation, the blocked blood causes headaches: “You have headaches during the day… people said that the headaches are caused by the blood that does not come out because of not menstruating”.

Vuyo eventually decided she could no longer tolerate the headaches: “I had persistent headache and decided to stop using the injection”, electing to switch to the implant: “I was supposed to choose from the implant or those injections… I remember that she said that one is for five years, so I asked for the implant… I went to the clinic and told them about my decision and asked them to give me the implant”. Unfortunately, after using the implant for a short period, Vuyo realised it wasn’t working well for her: “I had the implant for 4 months and I got very sick and even lost weight… I continued menstruating sometimes two times in a month and I could see that it was not good for my body”. She explains that while the implant works for some people, it was not good for her: “The implant works for some; they do not have challenges with it… Some menstruate for a long time and others do not menstruate… Others do gain weight, and it is good for them… I used to hear people talking, and I also heard at the clinic that the implant is good because it will take three years before coming again at the clinic, and that is what I liked about the implant but unfortunately it was not good for my body”.

After deciding the implant wasn’t working for her, Vuyo narrates: “I went back to the clinic to ask them to remove the implant… they were hesitant to remove it because it was supposed to last for years”. But Vuyo insisted, citing knowledge of her reproductive rights: “I begged them to remove it because **we all have rights**, and they removed it”. Vuyo then decided to use the 3 month injection: “they gave me the three-month injection because I did not want to get pregnant”.

In concluding, Vuyo asserts knowledge of her reproductive rights, explaining that there are many methods of contraceptives, and it is up to her to find the one that suits her the most: “**I will go to the clinic and ask the nurses about the contraceptive methods to use and I will change them if they are not good for my body until I find the best method that will work for me so as to protect myself from getting pregnant**”.

### Refi’s contraceptive journey

Refi, from Moretele, is 22 years old. Refi starts the story of her contraceptive journey by narrating that she went to the clinic because “I wanted to use contraception… but I was scared”. Refi underwent a routine pregnancy test at the clinic: “unfortunately I was pregnant… they started to judge me… I told them that I wanted to do termination… I did not want a child”. In Refi’s words, she was told that she “bleeds too much”, and was refused an abortion, instead being advised that she should have a follow-up clinic visit to have her uterus “cleaned” to avoid future miscarriages.

Soon after the baby was born, Refi started using Depo-Provera: “I gave birth in September then I used Depo in January… I used it for the entire year after that”. Refi shared her perception of Depo-Provera’s efficacy: “I liked it because it is strong, even if you skipped it is not easy for you to get pregnant because it is very strong”. Refi was told that despite being labelled as a 3-month injection, ‘Depo’ actually lasts longer: “It does not expire fast in your body, and even at the clinic they tell you that it stays longer in your body, it lasts for a period of four months while it is still in your system”. However, Refi explains her belief that the efficacy of the method depends on the individual: “Depo… depends on a person, how strong are you, someone might tell you that I used to use Depo but I am pregnant, the other one might tell you that it’s been a while using Depo and I don’t get pregnant… it depends… how weak or how strong you are… We do not react the same way… **we girls**,** our bodies are not the same**… You can react well to it (Depo injection)… you might get your periods well, without you gaining weight, but when I use it I might gain weight… and get my periods for a long time”. Side effects that Refi experienced included disrupted menses and weight gain: “I had bad luck of having my periods in a crazy way… you gain weight… crave things”.

Refi also shared her experiences of choosing and using the 2 month injection: “About Nur-Isterate (I learned about it) from the lessons we attend at the clinic… they teach us about it and **before they can inject you… you ask them ‘can you please tell me how it works…?**’… it’s an injection that you get for two months… mostly they use it for school kids… (because) it is not that strong compared to Depo”. In Refi’s understanding, Depo-Provera can damage the uterus and therefore AGYW who have not yet had children are advised to use Nur-Isterate, which is safer. One disadvantage with Nur-Isterate is that it doesn’t last as long: “It goes out of the system fast, so immediately when they booked you for a specific date, they say come at this date which means by that time it will already be out of your system… you must go back to get the injection”.

When Refi was using Nur-Isterate, she did not experience any side effects or changes in menses: “I did not use Nur-Isterate for a long time and then during the month that I used it I did not gain weight… nothing happened… I was just normal… my periods were fine… I only used it for two months.” The reason Refi discontinued using the Nur-Isterate is that she disliked having to go to the clinic every two months for a new injection and would skip or postpone her clinic visits: “I was scared of the clinic… queues… you wait there at the clinic… God you wait… you must go early in the morning… and it’s far… Honestly I don’t want to lie, I was scared of the clinic”. Refi explains that she would keep postponing her clinic visits: “when I had to go there especially on my appointment I used to feel like ‘Oh God I’ll go tomorrow”.

Refi explains that her reluctance to go to the clinic was a barrier to continuing with injectables. Since she’d missed clinic visits previously she was advised that they wouldn’t give her another injection: “I skipped clinic for almost one month, then when I went back they told me that they won’t give me the injection because I skip clinic…[laughing]”. Refi explains that the healthcare providers told her: “I must sit down and think properly whether I insert implant because it’s three years or I can leave”. She admits that she’d given false excuses in the past about why she missed clinic visits: “I was giving them unrealistic excuses saying ‘I forgot’. So they decided that I should sit down there and decide to insert implant… they told me… it’s for three years and you will remove it at a certain date, and then there’s a card that they give to you without that card they will not remove it”. Refi explains her decision-making process: “I am scared of the clinic… so I (decided I) will just do it… I realised that it cut the baggage of going to the clinic… now when I go to the clinic I go for a reason… Then I said ‘okay let me insert implant hence **it’s shortening my journey**’… for three years… let me insert implant”. Refi narrates that she was strongly encouraged to choose the implant by the healthcare providers: “at the clinic they decided for me, they inserted implant on me”.

Refi shares her view that the implant is effective for preventing pregnancy: “The implant does work well…without knowing much because **our bodies are not the same**, but I never got pregnant, it is good”. The best aspect of the implant in Refi’s view is that it saves you from having to go to the clinic: “Implant it’s something that works for about 3 years within someone’s body, whereby a person can remove it after three years… it works to prevent you from getting pregnant… what I like about it is that you will not go to the clinic over and over”. However, the downside of the implant in Refi’s view, is that it causes hunger and food cravings, which lead to weight gain: “it will make you gain weight… I was not weighing this much before! [laughing]… I was not craving things the way I do since I inserted implant, and I was not eating as much… Implant controls me, after I eat I go to bed [laughing]… when you wake up in bed it’s food (you think about)… you must always eat… you must feed cravings, if you’re craving certain things you must make sure that you get them”.

An additional side effect that Refi experienced since using the implant was disrupted and irregular menstrual bleeding: “(Bad things) about implant… I go on my periods for longer… and even though they’re not heavy, you see spotting… I can go a month spotting but when my period comes, it will be a heavy… heavy flow… and I can skip months without my periods, I can even complete six months without having my periods”. These disruptions to her menses concern her, as she believes you need to bleed for the ‘dirt’ to come out of the uterus: “It is not healthy when you do not get your periods because that blood is dirt… it must come out”. In addition, Refi believes that the implant damages the womb and causes infertility, which will jeopardise her marriage prospects: “Implant at the end will cause damage whereby I won’t have children anymore… You will find that my womb is damaged… it will cause me trouble in future [laughing] …In future if I get married and they say ‘have a child’… how are you going to work it out?”.

As a result of these fears, Refi wanted to get the implant removed but she was told by the healthcare providers that she should wait for the full three year period. She was informed that she could go to a private doctor if she wanted it removed earlier. She was reluctant to do so because of the costs: “They refused to removed it… they said I must wait and complete my three years… unless I go to the private doctor to remove it. But… they want money… so I have to pay”.

Refi plans to use the injection after this implant is finished: “(I won’t decide to use implant in the future). I don’t want it anymore plus my periods for a month… I will use injection when I remove implant”. However she explains that she wants to take a break from contraceptives first, to allow her body to detox from the chemicals in the implant: “I will stay for some time and clean (detox) myself to remove that chemical… I will clean my stomach and clean everything… that’s when I will start using injection properly, and use injection well”.

In concluding, Refi articulates an awareness of her reproductive rights and that she can choose the method that suits her best: “now we live in technology times, before you insert… make a decision, do research about what you want to use… log-in to Google, they will provide you with information on how Depo works, what are the side effects, because sometimes at the clinic I feel like… **they force us to use contraceptive just for our safety of not getting pregnant without knowing how will those things affect our body**,** how will we react to them**. They are able to tell you ‘use Depo’, when you get home you don’t respond well to Depo… so before you use contraceptives investigate first about that contraceptive to see how it is, and will you react to it… **at the clinic they do not give full information**… **I will look at them (methods)… all of them have their consequences… you look at which ones… are suitable for your body**”.

### Dinah’s contraceptive journey

Dinah is aged 16, and is from Moretele. At the time of being interviewed, Dinah has not been using contraceptives for long. Dinah explains how she felt when she first went to access contraceptives, accompanied by her friend: “At first I was scared… I was with my best friend and… she scared me… she was holding my hand and she was like ‘it’s painful… she was making me nervous”. Dinah was particularly concerned about side effects, most notably infertility: “I was like okay, I did ask about this… a lot about side effects… if I’m a grown up and it’s time for me to have babies wouldn’t it affect me somehow… it just burns out eggs and ovulation time for me not to catch up pregnancy”.

After considering her options, Dinah specifically chose not to take oral contraceptives as she was worried that people might think she was taking antiretrovirals (ARVs) indicating she had HIV, and was worried about potential accusations and stigma: “you know taking pills, sometimes people ask… ‘you’re taking pills what kind of pills are those?’… they’ll be too judgmental… and some make fun… they might be like okay ‘You’re taking ARVs’… so some of us don’t take it light, we take it heavy… offensive”. Dinah selected a contraceptive method she thought would suit her: “the injection… for three months… I thought… it’s a once-off thing… you just have to go back… if it’s your due date… I chose this method because I thought it was the best for me… what I liked about this is that it takes for a long time and it doesn’t affect your period cycle”. She had faith that if she did experience side effects, she would be assisted: “if you do get affected you go back and tell them that your (menstrual) cycle is not working properly, so they give you another medication… if it gives you mood swings… you can go back and tell them that so that they can give another medicine… to cure that”. Dinah believes that if you experience side effects, it’s a sign that “your body is working the way it (was supposed to)… responding”. She says that it takes a while to get used to the injection: “when you’re first using it, it does that (causes side effects)… you get used to it, your body eases up”. Dinah explains that she has experienced some side effects: “my appetite is bigger… it makes you have appetite… you’re always eating”.

Dinah plans to continue using contraceptives as she strongly believes in the importance of doing so: **“because I am taking part in reducing teenage pregnancy in the world**… (at first) I was a little bit scared and nervous… but I was like okay **I’m doing this for me… for my body, if I know that I’m going to be sexually active, I have to protect myself**”. Dinah noted that for her, a major benefit of taking contraceptives is that “you don’t worry that you have to take morning after pill, go to the clinic or something like that… it’s an advantage for you using them”.

### Lebo’s contraceptive journey

Lebo is 19 years old, and lives in Moretele. Lebo is currently using the contraceptive implant. Before getting the implant inserted, she consulted her mother to seek permission, even though she feared her mother’s reaction to her wanting to use contraceptives: “I was scared because I had a fear that she will ask me why I wanted implant… I did not know how I will be able to respond to her… because I was embarrassed… My mother?… she felt like beating me hard… it was not easy… (but) then my mother said ‘If you want implant you can go, I am giving you permission’… I was scared and I was not sure but I took my mother’s decision, because she was able to sit me down and talk about what was happening… so I inserted implant”.

At first Lebo didn’t know much about the implant and found the concept of it frightening compared to the more familiar injection: “I was scared… I didn’t know what kind of a thing is implant because they explained to me… that there’s an implant, it can last for three years – what kind of a thing is this which can last for three years?… we are used to Nur-Isterate and Depo”. Lebo’s fear of the implant was also due to what she’d heard about it: “my friends said that implant is not okay… it moves (inside)… I was scared… (because) other people spoke endless things… They made me feel like I want but don’t want to, I was scared”. However despite her anxiety, and with the support of her mother, Lebo went ahead with her decision as she wanted to prevent pregnancy and had faith that the clinic would have warned her if it was dangerous: “When I was accessing implant right, I was scared but… then I realized that there’s nothing wrong about it… I realized that I don’t remember at the clinic being told it moves or anything… I realised that it’s alright, I’ll remove it on the due year when I see that I’m ready and I want to have a child”.

Lebo explains her reasoning for choosing the implant was that she was too busy to go to the clinic regularly and didn’t want to be distracted from her education: “I was doing grade 10, and I was no longer able to come home and then go to the clinic to prevent (get contraceptives) because I have a job to do at home… I decided because there’s this implant for three years, it can save me from travelling, enabling me to do my chores at home, I can also concentrate on school… it will help me to save time, to concentrate and… help my parents here at home”.

In addition to viewing the implant as a time-saver, Lebo also believed the implant to be more effective than other methods, as she’d heard about a friend getting pregnant whilst using the injection: “Implant I think it’s the best one, because my friend told me that she was pregnant and she was using injection… I was shocked… so I decided no, let me use implant, because my friend got pregnant while using this thing Nur-Isterate”. Lebo’s shock was due to the faith she had in the efficacy of contraceptives: “It hurt me because these things contraceptives I trusted them… I couldn’t listen to her story because I was scared and I was hurting… I was asking myself… how come someone got pregnant while using Nur-Isterate?”.

Lebo wanted to show her peers that their fears of the implant were unfounded: “I wanted to prove a point… I wanted them to stop talking about things that they have never experienced… I told them that for me implant is treating me well and it doesn’t move, so they shouldn’t tell people lies”. Lebo believes that spreading misinformation deters girls from using contraceptives and preventing pregnancies: “they should stop making other girls scared of going to the clinics, because the more they become scared of going to the clinic it’s the more the pregnancy increases” … you need to think for other girls… someone will go and have sex and get pregnant… just because she is afraid to use implant”.

Lebo did experience some side effects after first having the implant inserted: “The first month after using it, I got agitated a bit, got dizzy… first month I struggled bit”. But now Lebo is satisfied with her choice to use the implant: “I feel very happy (using implant), because I am reacting well to it, it doesn’t cause any diseases… I’m just well in my body… I even forget that I have it in my body”. Lebo concludes that using the implant will enable her to complete her education and not place extra pressure on an already struggling household: “Implant will keep me for three years, I will complete school while it is still here… I won’t bring a child while I can see the situation we have at home, and I react well to it, I’m happy while I have it”.

## Discussion

Recognising contraceptive use as a continuous undulating journey spanning the reproductive life, entailing on-going decision-making that is influenced by a wide range of factors, rather than a once-off medical event, is critical. These findings, inclusive of adolescent girls’ and young women’s narratives, show the complexity and contextual nature of contraceptive decision-making and the lived experiences of contraceptive use that AGYW navigate. The provision of, and support for the use of contraceptive methods for South African AGYW, particularly school-going adolescents in hard-to-reach communities with high rates of pregnancy, needs to be responsive to the realities of their everyday lives. Understanding how AGYW perceive different contraceptives, and the factors they consider when making decisions about which method to choose is critical in enhancing sexual and reproductive service provision for AGYW, and in informing policy and programming. As illustrated by the narratives of AGYW contraceptive journeys, the lived realities and embodied experiences of AGYW as they choose, use, and access contraceptives are complex and multi-faceted.

Respondents in our study shared their views and beliefs on contraceptive implants, injectables, and oral daily pills. In the qualitative data, convenience was considered an important characteristic of long-acting contraceptive methods, with not having to remember to take a daily pill regarded as beneficial. Consequently, perceived negative attributes of a contraceptive method included being inconvenient, having to take a pill daily, having to go to the clinic regularly, having a foreign object inserted in your body, and having something ‘inserted/removed’ by a healthcare provider. When AGYW make decisions about which contraceptive method to choose, they consider various characteristics, perceived pros and cons of each method. The way in which AGYW perceive the efficacy of a method, possible side effects or safety concerns, convenience of use, and popularity amongst peers, all play a part in influencing decision-making around which contraceptive method to choose, use, and continue using. The subjective embodied experiences that contraceptive users have of specific methods, and the meanings they imbue those methods with, impact their willingness to maintain consistent and continued use [1]. AGYW’s understandings, preferences and valued characteristics of contraceptive methods are fluid and subject to shifts and modifications, reflected in fluctuations in method selection and use [1, 22].

Our survey findings indicate that the injection was considered the best method, followed by the implant and the pill. Amongst survey respondents, the majority believed the injection to be a good contraceptive method option, and approximately half believed the implant to be a good contraceptive option. According to data from South African Demographic Health Surveys, progestin-only injectables are the most popular contraceptive method amongst AGYW, due to their convenience, high acceptability among AGYW and health providers, and cost-effectiveness [2]. Less than half of all survey respondents in our study believed the daily oral pill to be a good option, despite the fact that it was regarded as having the least side effects. In qualitative interviews, respondents cited concerns that taking a daily pill may lead others to mistakenly assume it was HIV treatment, causing possible stigma. Respondents also suggested that since AGYW tend to be busy and forgetful, remembering to take a pill every day can be challenging. The view that taking a pill every day is onerous and challenging is not a new one, as evidenced by prior research conducted in South Africa [7]. There was minimal qualitative data on AGYW’s views of and experiences using IUDs, with survey data indicating that less than 6% of AGYW had been told about the IUD by a healthcare worker. Evidence from the global context highlights the importance of a product’s perceived ease of use and convenience in its acceptability and popularity [4, 23].

For AGYW, one major perceived attractive attribute of a contraceptive method includes a method being long-lasting, which means not having to go to a clinic regularly, considered to be onerous, time consuming, and risking being seen or judged. A method being accessible at a pharmacy instead of a clinic, or not requiring a health professional’s intervention, were also regarded positively. Reluctance for frequent clinic visits was one of the main factors influencing AGYW contraceptive decision-making in our study. Refi’s narrative in particular illustrated the lengths that some AGYW will go to in order to avoid going to the clinic, intentionally missing clinic appointments, thereby failing to adhere to the 2 monthly injection. As well evidenced, there are many barriers to AGYW accessing health services, especially those provided through government clinics. Therefore, a major benefit of the implant, as perceived by AGYW in our study, is the fact that it’s a long-acting method, and does not require the user to go to the clinic more than once every three years. Evidence from across the globe suggests that women consider it a negative attribute of a contraceptive method, if its use requires more regular, or closer engagement with formal healthcare settings [22, 24, 25]. A study amongst women in South Africa also found that longer-acting methods were valued for the decreased need to attend clinics, which often have long waiting times [7].

Changes or disruptions to the menstrual cycle or menstrual bleeding were a key concern of AGYW in using contraceptives. Respondents described perceived negative effects of contraceptive methods on their menses, including spotting, heavy, prolonged or irregular periods, or ‘blocked’ blood flow. In our survey data, despite 44% of AGYW in the older age group believing the implant to cause irregular bleeding, 37% believed it to be safe to use, and half believed it to be a good method option for AGYW. In our qualitative data, the narrative shared by Vuyo illustrated the concern around the perceived negative effects of any change or cessation in menstrual bleeding. Vuyo believed that the ‘dirt’ (menstrual blood) needs to come out, and if blocked, will cause headaches. This conception of the crucial role that menstrual blood flow has in cleansing the body and womb is common across the sub-Saharan African region; fears related to blocked menstrual blood flow have been shown to be a key barrier in women’s willingness to use hormonal contraceptives [26, 27]. Global evidence suggests that the effect of hormonal contraceptives on menstruation, is a key factor in women’s contraceptive decision-making, particularly the decision to discontinue a method [4, 15, 28]. Evidence from South Africa suggests that amongst women using implants and injectable contraceptives, there is a high rate of discontinuation specifically related to experiences of changes in menstrual bleeding [27, 28].

As illustrated in the contraceptive journey narratives, in many cases AGYW stopped using contraceptives due to embodied experiences, and feared side effects. Despite the general popularity of implants and injections, participants in qualitative interviews cited feared side effects of long-acting contraceptive methods, most notably concerns around long-term infertility, changes to the menstrual cycle, and headaches. In the survey, more than half of AGYW expressed safety concerns around contraceptives, with only 45% believing the injection was safe, 37% believing the pill was safe, and only 30% believing the implant was safe. The lack of trust in the safety of contraceptives amongst AGYW is likely to undermine acceptability and uptake. Amongst qualitative respondents, it was believed that the implant caused infertility, whereas the oral daily contraceptive pill was safer in this regard. In the survey, a quarter of respondents believed that the implant may negatively impact fertility. Evidence shows that the belief that hormonal contraceptives cause infertility is widespread [5, 11, 22]. Weight change was also a concern, however some AGYW viewed weight gain positively, whereas others regarded weight loss negatively. Increased appetite and hunger were viewed as problematic particularly for those in resource restricted households where food was not plentiful. In the survey data, more than half of AGYW believed the injection makes the body change in unpleasant ways.

Qualitative data revealed the belief that it was beneficial to take a break from specific methods, to allow the body to ‘detox’ from the chemicals. Prior research has also found that AGYW believe it important to take breaks between contraceptive injections to give the body a rest [29]. Inconsistent use and discontinuation of contraceptive methods, particularly the implant, remains a key concern in South Africa [15, 30–32]. Evident in the AGYW narratives were also descriptions of the choice to stop using a specific method, rather than stopping contraception all together. This is referred to as ‘method-specific discontinuation’, which for programmatic reasons should be distinguished from contraceptive discontinuation [21]. Although method-specific discontinuation implies that a user is making informed agentic choices around finding a method most suitable for her, method switching can, if not adequately supported and counselled, result in unintended pregnancy, as evident in the AGYW narratives.

Familiarity with drug delivery mechanisms was also a factor, with injections and oral tablets regarded as more familiar than subcutaneous implants. Uncertainty around novel technologies and methods has been shown to serve as a barrier to use of modern long-acting contraceptive methods [1]. Additionally, some AGYW stated that they would be scared of using an implant, which might be seen as invasive and alien. A method being discrete (non-visible or tactile) was also seen as an attractive characteristic in a contraceptive method by respondents in our study. The fact that an implant may be actually visible in the arm concerned some respondents. The physical placement of a contraceptive device or method within the body has also been shown to be a key concern amongst AGYW when selecting a method [1]. Previous evidence from South Africa supports the notion that the ‘palpability’ of implants is a concern amongst AGYW [8]. The current modality of subcutaneous implants available are not dissolvable or biodegradable and require surgical removal by a healthcare provider, which may reduce acceptability amongst AGYW [8]. Qualitative findings from our study showed that one barrier to AGYW acceptability of the implant related to fears of implant theft. This narrative has emerged in previous research from South Africa, demonstrating the ways in which beliefs about contraceptive delivery mechanisms impact user acceptability and choices [30, 33].

Method efficacy was viewed as an important attribute by AGYW respondents, citing the ‘strength’ of a method as a consideration. Additionally, it was acknowledged that ‘not all bodies are the same’, and therefore some methods may be effective for some people, but not work for others. In the AGYW narratives, there were accounts of AGYW getting pregnant whilst using a contraceptive, although in some cases this was allegedly due to user negligence. It was suggested that AGYW are at risk of getting pregnant with the injection because they fail to follow up treatment. Generally, a contraceptive product’s perceived effectiveness and reliability will determine its acceptability amongst users [4, 22, 34]. There is some indication that amongst South African AGYW, knowledge around reproductive system physiology, and contraceptive methods’ mechanisms of action is lacking [1, 11]. Perceptions of and meanings ascribed to side effects are contextual and socio-culturally informed, particularly those relating to fears of infertility, and are often dismissed by biomedical health practitioners as ‘myths and misconceptions’ [21]. However, whether or not the side effects of contraceptives are clinically ‘provable’ or objective, it is their lived reality and embodied experience which will impact AGYW willingness to use a method. If an individual perceives a somatic sensation or embodied experience to be connected to a contraceptive method, they are likely to discontinue using it [1, 7]. Given that perceived and somatically experienced side effects are the primary reason for method discontinuation, it is critical to consider these in order to ensure the provision of acceptable, appropriate, respectful and comprehensive contraception services [15, 21].

Respondents described the centrality of peers and friends in decision-making about contraceptives. The reputation that different contraceptive methods have amongst peers play a key role in determining AGYW willingness to try them. Peers and friends exert strong influence on adolescents and young people’s behaviour and decision-making. As a consequence, the perspectives and narratives of peers around contraceptive method choices, as well as perceptions of peers’ use of contraceptives, play a large part in AGYW contraceptive decision-making [5, 11]. AGYW’s own understandings and experiences of side effects, and peers’ discourse of side effects, are likely to have a stronger influence on AGYW decision-making around contraceptive methods choice than healthcare providers’ advice. There is some evidence to suggest that women are more likely to select a specific contraceptive method if others in her social network are also using it, and often decide upon a specific method prior to seeking healthcare services based on information received from peers [5].

As the qualitative narratives in our study show, AGYW do also consider the advice of healthcare providers, however, they sometimes felt that their agency was constrained by healthcare providers in cases where their preferences were not considered or respected. Moreover, some respondents felt that healthcare providers failed to provide comprehensive explanations about possible side effects. In the survey, almost a quarter of AGYW reported that they had been steered or pushed towards a specific method, and only half reported that the health worker had checked whether she was happy with her previous method. Reporting in the survey suggested that health workers do not provide equal information on all methods available, with more AGYW reporting they’d been told about injectables over any other method, followed by the implant, the pill, and lastly very few being told about the IUD. For survey participants who reported that they had been steered towards a specific method, it was mostly towards the injection or implant. Previous evidence has highlighted similar sentiments amongst South African AGYW who felt that their freedom of choice was constrained in selecting a contraceptive method, and that parents and healthcare providers often choose for them, with injectables and the implant deemed as most suitable [12, 13].

These findings bolster the argument that contraceptive users require guidance from healthcare providers, who can assist them in exploring their individual values and preferences, but in the end desire agency in making a final informed decision of which method to use [4]. The narrative journeys in our study illustrate the ways in which AGYW contraceptive agency is constrained by the limited information they are provided with, and by the guidelines to which health workers adhere. This was evident for example in the way in which Refi wanted to get her implant removed but was told by the healthcare providers that she had to wait the full three-year period. Our findings build on evidence suggesting that healthcare providers do at times restrict women’s contraceptive method choices based on their own biases and personal judgements about suitability; in particular the agency of AGYW to make contraceptive choices is often undermined due to personal beliefs and attitudes towards providing contraceptives to adolescents [12, 35, 36]. Even when providers follow clinical guidelines in prescribing contraceptives, these standardised guidelines may fail to consider women’s individual needs, preferences and contexts [35]. A reproductive rights approach recognises that each individual with reproductive capacity should be afforded agency and supported in making decisions throughout their reproductive life course [6]. In Vuyo’s narrative, she articulates an awareness of her reproductive rights, and the agency she has in her contraceptive decision-making. To date, adolescent SRH interventions have focused largely on expanding access to and improving quality of health care services, and enhancing AGYW’s SRH knowledge [37]. Our findings add to an emerging evidence base suggesting that there is a critical need to simultaneously enhance the agency and autonomy of AGYW to make informed decisions around their SRH [38, 39]. Given that AGYW with higher levels of agency, self-efficacy and communication skills can better express their SRH concerns and seek SRH information, interventions designed to foster positive SRH outcomes among AGYW should aim to develop agency as a core programme component [37]. Through sharing their contraceptive narratives, AGYW are afforded the opportunity to articulate their individual experiences and journeys, thus facilitating more responsive and appropriate service provision [14].

One of the strengths of this study is the mixed-methods design. Both quantitative and qualitative research was conducted among AGYW from the same 14 schools in the Moretele and Newcastle areas. However, a limitation of the quantitative data is that it is only generalisable to AGYW from these schools and not representative of all AGYW in the two study districts or in South Africa. Nevertheless, it provides valuable insights on school-going young women located in hard-to-reach communities with high rates of poverty and unemployment and unintended pregnancies. A limitation of the qualitative research is that contraceptive journeys as such were not the initial focus of enquiry, but were nested within a broader exploration of young women’s needs and health contexts, therefore the depth of investigation into each individual journey was limited. However, with the narrative journey participants, this lack of depth was mitigated to an extent through returning to the participants for the member-checking process.

## Conclusions and implications

As our findings on contraceptive journeys illustrate, the needs and preferences of AGYW are fluid, shifting and changing depending on context, beliefs, fears of side effects and somatic lived experiences. These findings add to the evidence base supporting the notion that contraceptive journeys are complex and dynamic, and do not follow a linear process of decision-making and use. Unlike many other health interventions, pregnancy prevention is an on-going iterative process of decision-making, subject to influence by a multitude of factors and actors, that shift and change throughout the multiple decades of the reproductive life course [6, 14]. The narratives of AGYW in our study build upon evidence showing that contraceptive experiences of each individual are cumulative, temporal, and comprise a continuum of identification of need, method choice, initiation, use, discontinuation, method switching and on-going circular decision-making influenced by a wide range of factors [6, 14].

Framed within a reproductive justice and rights based discourse, these findings suggest that in South Africa there is a need to improve the responsiveness of contraceptive service provision in order to ensure that the changing contexts, needs and preferences of users are considered, so that pregnancy prevention needs are met [1]. In order to maximise the use of contraceptives amongst South African AGYW, it is necessary to provide a range of method options, and comprehensive information about each, so that users can make informed decisions to choose and use the method that best suits their needs, preferences, lived reality and experiences at that moment in their life [22, 28]. According to the reproductive justice framework, patient autonomy and choice should be the principial determining factor in the selection of contraceptive method [9, 39]. Whilst our data shows that AGYW generally prioritise the views, opinions and advice from peers over that from health care providers, AGYW still need to be supported in choosing, and using the contraceptive method that best suits their needs and context at any particular time [4]. It is also important to recognise that these needs and contexts will shift and change, as will the most suitable contraceptive method, and therefore method choice and suitability should be discussed at each encounter [4, 27]. Contraceptive discontinuation can be mitigated by facilitating user choices and supporting method switching when necessary [28, 31]. What this requires is equipping SRH healthcare providers with the skills and capacity to assess each individual AGYW’s values, needs and preferences, and to provide the necessary information so that the user can make decisions that will maximise their ability to obtain their sexual and reproductive goals [4].

Ensuring the availability and accessibility of accurate SRH and contraceptive information through interventions, programmes and educational campaigns is critical [11]. Additionally, healthcare providers play a critical role in providing comprehensive contraceptive counselling and information, to support contraceptive decision-making and encourage method continuation or switching [40]. It is crucial that AGYW are provided with comprehensive information around possible changes or method induced amenorrhea to allay fears and concerns around menstrual disruptions, reduce the likelihood of method discontinuation, and thereby ensure continuous contraceptive coverage [27]. Information and counselling around side effects need to be context specific and responsive. For example, providing explanations on the difference between infertility and contraception-induced delay in return to fertility may go some way to addressing misconceptions [41]. Since the perceptions, beliefs, and embodied experiences of AGYW using contraceptives are likely to be more salient in contraceptive decision-making than biomedical advice, it is critical to validate these and address concerns in contraceptive counselling [1]. The provision of contraceptive counselling based on the reproductive justice framework is imperative in order to ensure equitable access to contraceptive methods for all AGYW who wish to prevent pregnancy [9].

## Data Availability

Availability of data and materials: The datasets used and/or analysed during the current study are available from the corresponding author on reasonable request.

## Footnotes

1 The South African National Department of Health guidelines do not stipulate that postpartum contraception is compulsory, do outline that postpartum women should be encouraged to choose a contraceptive method, some of which can be provided immediately after birth. The guidelines state that as with all contraception provision, postpartum contraception needs to be framed by rights-based, client-centred, quality care (NDOH, 2019).
